# Supplementary material for: Mitochondrial Respiratory Defect Enhances Hepatoma Cell Invasiveness via STAT3/NFE2L1/STX12 Axis
Source: Cancers (Basel). 2020 Sep 15;12(9):2632. doi: 10.3390/cancers12092632 (PMC7565734; doi:10.3390/cancers12092632)
Supplement: Supplementary file 1 [file cancers-12-02632-s001.pdf]

cancers

*Supplementary Materials*

# Mitochondrial respiratory defect enhances hepatoma cell invasiveness via STAT3/NFE2L1/STX12 axis.

## Whole Blots for Western Blot analysis

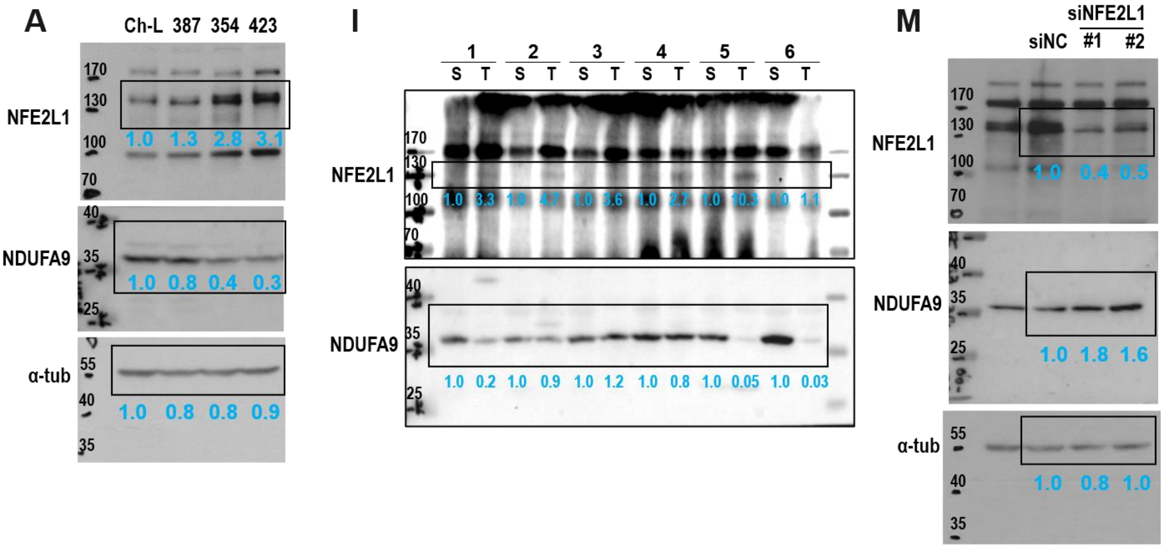


**Figure S1.** Whole Blots for Western Blot results of Figure 1

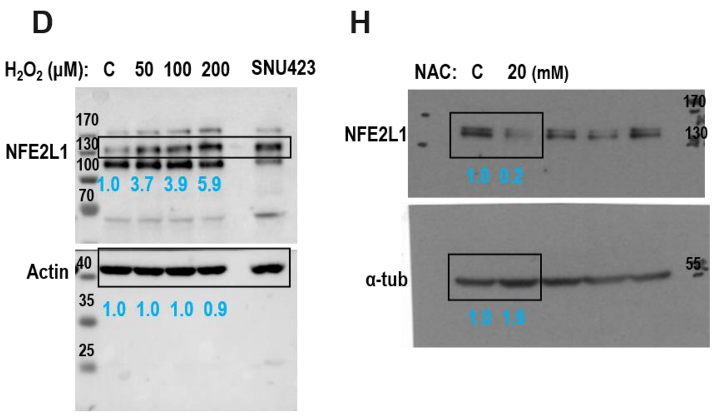


**Figure S2.** Whole Blots for Western Blot results of Figure 2

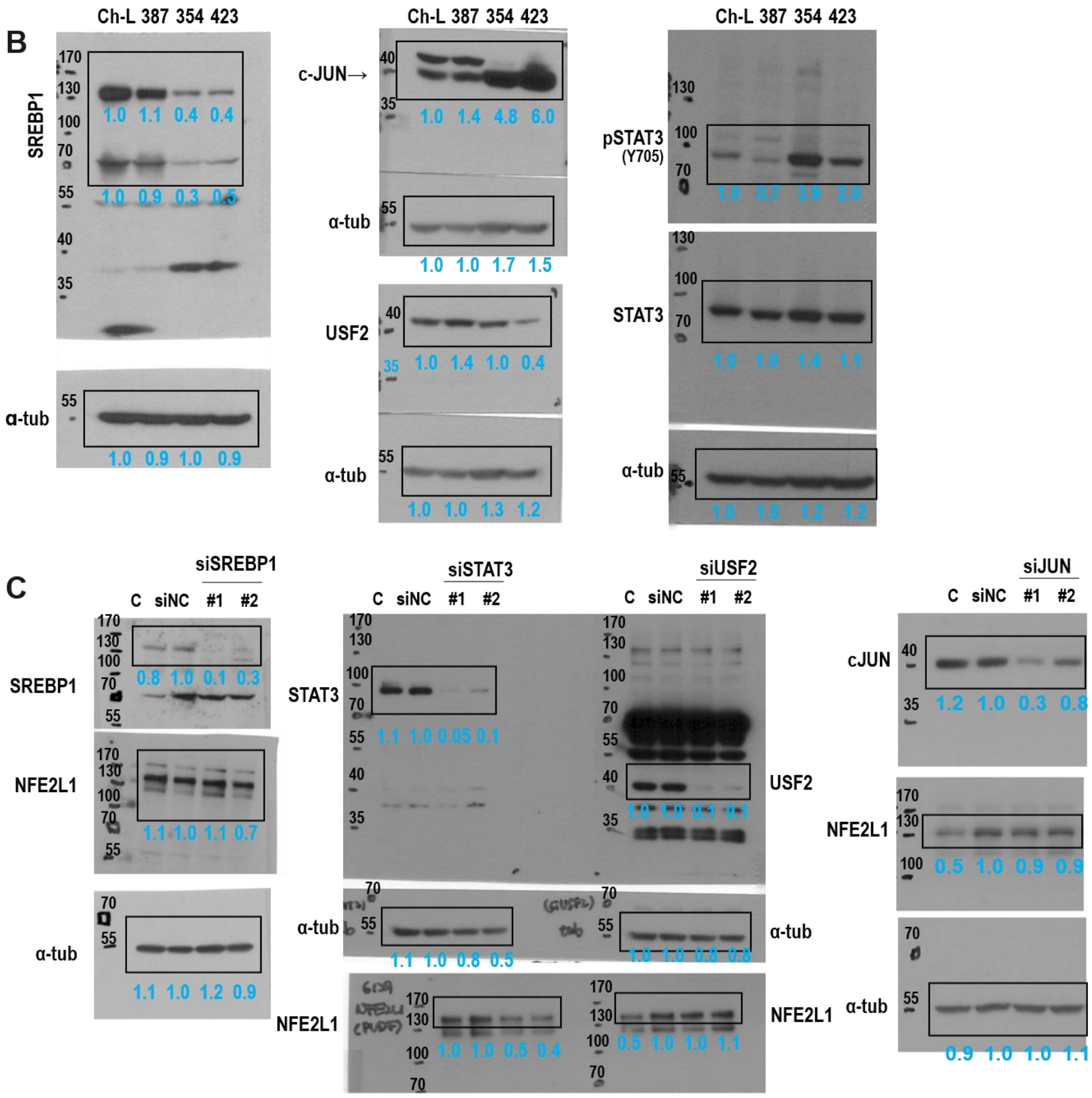


**Figure S3.** Whole Blots for Western Blot results of Figure 3

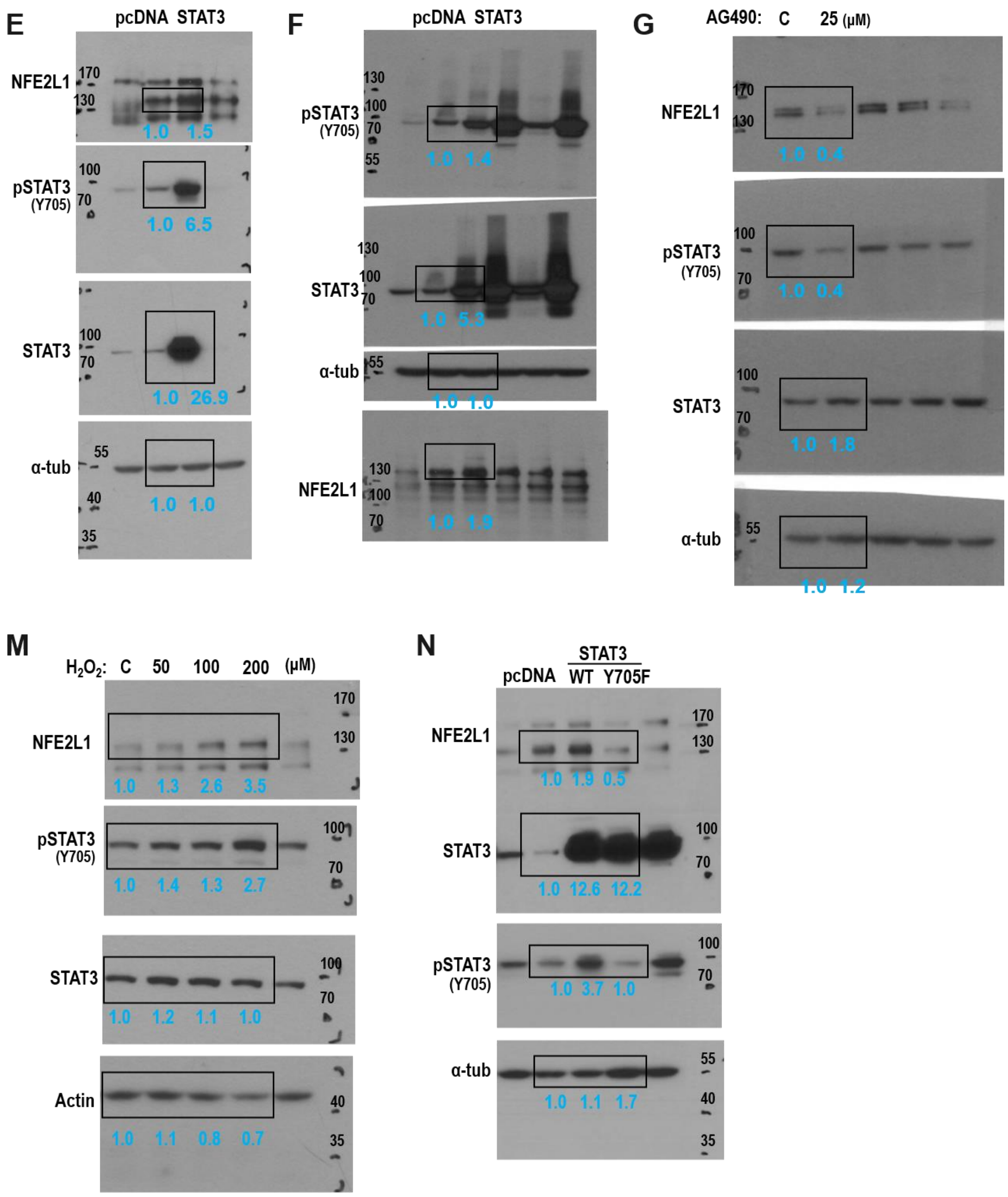


**Figure S4.** Whole Blots for Western Blot results of Figure 3 (continued)

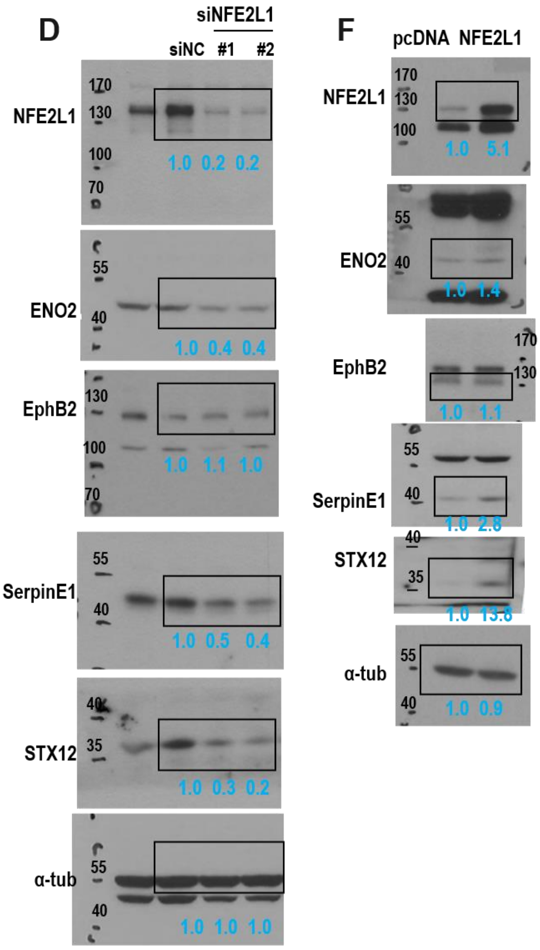


**Figure S5.** Whole Blots for Western Blot results of Figure 4

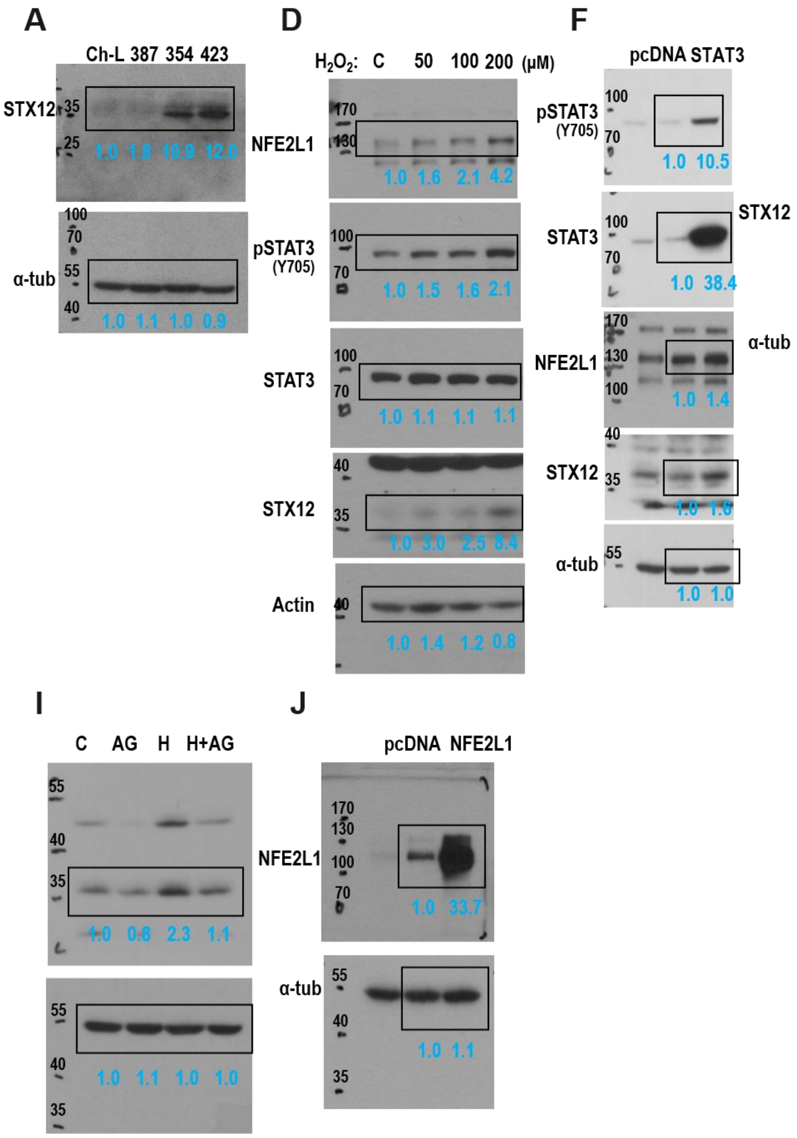


**Figure S6.** Whole Blots for Western Blot results of Figure 5

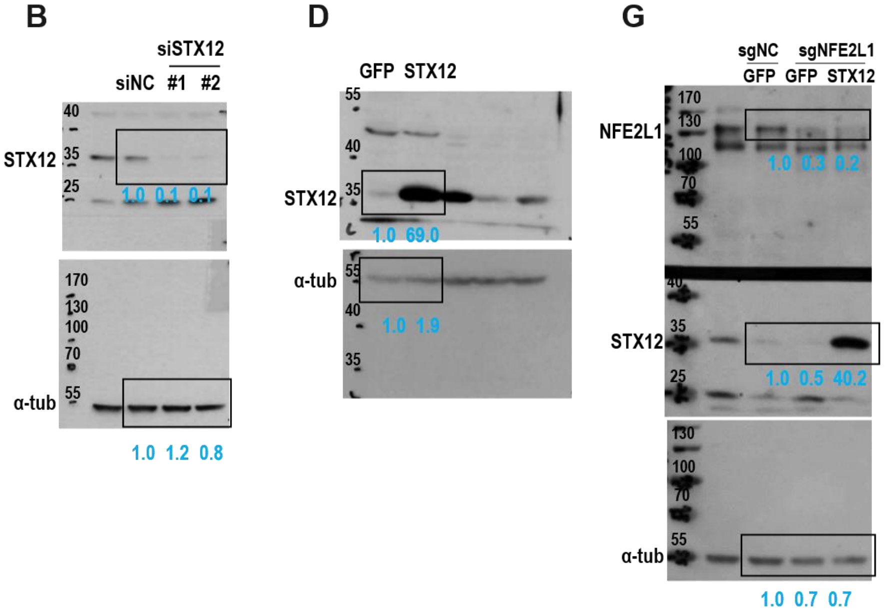


**Figure S7.** Whole Blots for Western Blot results of Figure 6

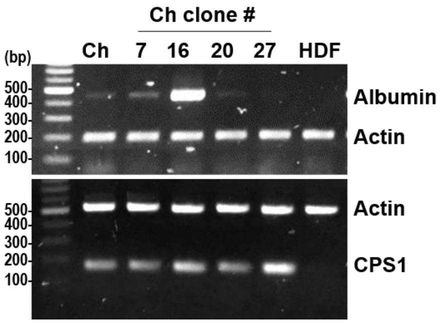


**Figure S8.** Isolation of Ch-L clones which harbors liver-specific gene phenotype. Chang cell clones were isolated by serial dilution and the 4 clones with liver cell characteristics were selected by monitoring albumin and carbamoyl phosphate synthetase 1 (CPS1) mRNA level by conventional RT-PCR. HDF (Human Diploid Fibroblast) cell used negative control of liver cell characteristics. The PCR primer sets were produced by Macrogen, Inc. (Seoul, Kore): albumin (5′-GGGCATGTTTTGTATGAAT and 5′-CTGTTCACCAAGGATTCTGT), CPS1 (5′-CAGCTCTTGCAGAACCACC and 5′-ACAGGCTGATTCTGCCCTC) and actin (for 191bp, 5′-CCTTCCTGGGCATGGAGTCCTGT and 5′-GGAGCAATGATCTTGATCTTC; for 518bp, 5′-GCACTCTTCCAGCCTTCCTT and 5′-CTGTCACCTTCACCGTTCCA).

© 2020 by the authors. Submitted for possible open access publication under the terms and conditions of the Creative Commons Attribution (CC BY) license (http://creativecommons.org/licenses/by/4.0/).
